# School health in Europe: a review of workforce expenditure across five countries

**DOI:** 10.1186/s12913-020-05077-w

**Published:** 2020-03-12

**Authors:** Simon van der Pol, Maarten J. Postma, Danielle E. M. C. Jansen

**Affiliations:** 1 Department of Health Sciences, University of Groningen, University Medical Center Groningen, Hanzeplein 1, 9713 GZ Groningen, the Netherlands; 2 Department of Economics, Econometrics and Finance, University of Groningen, Groningen, the Netherlands; 3 Department of Sociology, Interuniversity Center for Social Science Theory and Methodology (ICS), University of Groningen, Groningen, the Netherlands

**Keywords:** School health services, Child healthcare, Health system comparison, Health expenditure estimation

## Abstract

**Background:**

Most European countries have implemented a form of school health services (SHS) to provide young children and adolescents with various types of healthcare. No estimations on SHS expenditure for European countries have been published until now. We estimated SHS workforce expenditure in Europe, expected to serve as the main driver of school healthcare costs.

**Methods:**

Using two networks of experts on healthcare provision for children we contacted various country representatives to provide data on the number of professionals working in SHS and salaries. These data were used, together with publicly available data, to estimate annual SHS workforce expenditure on the national level.

**Results:**

We received sufficient data for five European countries, and estimated the SHS workforce expenditure. Nurses were the most widely reported professionals working in this field, followed by doctors and psychologists. Our SHS expenditure estimations ranged from €43,000 for Estonia to €195,300 in Norway (per 1000 pupils). For Norway, Estonia, Finland and Iceland, school nurses were the main drivers of SHS expenditure, mainly due to their large numbers, while in Austria, school doctors played the largest role in SHS expenditure.

**Conclusions:**

We estimated the spending on SHS workforce for five European countries, which comprises relatively minor parts of total healthcare spending (0.16 to 0.69%). Many questions regarding SHS spending in Europe remain, due to a general lack of data on national levels.

## Background

Children’s happiness and health are known to be important societal values [1, 2]. It is generally considered that lifetime health outcomes and socioeconomic status are largely determined by an appropriate and stable environment at the start of life [3]. This highlights the importance for society to invest in health equity in life’s early stages, that may very well result in rewards as a result of increased health and socioeconomic status. Not only should international human rights incentivise countries to invest in the health of children, but also this economic principle [4, 5]. School Health Services (SHS), offered in most European countries, educate children from a young age regarding the importance of their health, screen them for various illnesses and provide care for those in need [6].

Recently, SHS care was researched within the Horizon 2020 funded Models of Child Health Appraised (MOCHA) project [6–8]. The organization and composition and content of SHS were explored by sending out questionnaires to country agents, local experts in child and adolescent healthcare, across 30 European countries, of which 28 countries provided school healthcare [6]. In many European countries, direct medical care was found to be a part of SHS, including tasks like the management of chronically ill children and emergency care. In almost all countries, SHS comprised screenings, with a focus on height and weight, as well as vision, hearing and dental tests. Another focus in most countries was mental health promotion. Key components of most SHS included preventative care, with a particular focus on communicable diseases with vaccinations, infection control and hygiene surveillance. Preventive measures included vaccinations (21/28), referrals to other health professionals (22/28), infection control (19/28) and surveillance of the school’s hygiene (18/28, 6]. Education was a key area within SHS in many countries as well, such as sex education and the promotion of a healthy lifestyle [6].

Although the urgency for investing in prevention within the healthcare sector is obvious, in European countries expenditure on prevention programmes only accounted for 1–5% of total healthcare spending in 2016 [9]. SHS expenditure data in general are unknown and its workforce has not been mapped in many countries. For example, the Organisation for Economic Co-operation and Development (OECD), which collects data on national healthcare expenditure, does not report data on SHS spending [10]. One factor of this incompleteness is that some parts of SHS are paid through healthcare budgets and others through education budgets; also, in many countries the responsibility of SHS lies with both local and national governments [6]. Although data on the costs and cost-effectiveness are available for specific interventions in specific countries, the evidence for the costs and cost-effectiveness of the general SHS is limited [11], including the most basic interventions offered (such as screening programmes, education on hygiene and infection prevention).

No studies have been published detailing the costs on a system-wide level for European countries. For the United States, studies determining the cost-effectiveness of running a school-based health centre have been published [12–14]. For specific interventions within schools, European cost-effectiveness studies are available, considering topics like: obesity prevention [15, 16], healthy food programmes [17], dental care programmes [18], attention deficit hyperactivity disorder education [19], smoking cessation [20], sexually transmitted infections prevention [21] and school-based immunisation programmes [22, 23]. Many of these interventions are considered to be cost-effective with relatively low budgetary investments [15–23].

As part of our investigations within the context of MOCHA, we examined SHS expenditure and system-wide health effects and concluded data on this topic was lacking [6]. We therefore aim to further investigate SHS expenditure in Europe by estimating the money spent on the SHS workforce, which we expect to be the main driver of SHS costs. With this research, we estimate SHS workforce spending on the national level in countries which are able to provide the necessary data. Additionally, we identify gaps in current knowledge. Better insights into the costs of SHS allow valid cost-effectiveness analyses of SHS in the future, potentially further supporting its attractiveness.

## Methods

### Questionnaire

Building on the country agent network initiated for MOCHA [7] and the economic data collected in work package 3 of the project [6, 8], we sent out a questionnaire by email, focusing on the workforce and gross salary of the SHS workforce. This included all countries within the European Economic Area, except for Liechtenstein. The country agents were experts in the field of child healthcare in their respective countries and used indigenous sources to collect data within the MOCHA project. They were selected using a mixed-methods approach and trained by the lead researchers of MOCHA [7]. In case the country agent did not respond, we also contacted the contact listed on the European Union for School and University Health and Medicine (EUSUHM) website [24]. The responses were visualized using a flow chart. Informed consent to use the responses for this research project was acquired from the country agents in writing.

In the questionnaire (see supplementary files), we asked the workforce (numbers) and remuneration estimates of the following SHS professionals (which were identified in previous research [8]):

- School nurses
- School doctors
- Psychologists
- Social workers
- Dentists
- Physical therapists
- Healthcare assistants
- Supportive staff
- Others

For the workforce, questions were asked on the fulltime equivalents (FTEs), the total number of professionals and their type of employment (salaried and/or self-employed). We asked the average gross salary; if this was unknown, we asked the general salary of a professional with 10 years of working experience in the field of SHS.

### External data sources

From the MOCHA project, we knew which SHS professionals were working in which countries: if no numbers were reported in the questionnaire, but the professionals were reported to be working in SHS in previous research [6, 8], we considered data to be missing.

To be able to compare the data between countries, all financial data were converted into 2018 euros and corrected for the number of pupils within each country. From Eurostat we acquired the number of pupils (4–18-year olds) for each of the included countries [25]. The consumer price indexes of the various countries were used to incorporate inflation and we corrected differences in currency and purchasing power using purchasing power parities (PPPs) [26, 27]. Labour costs other than wages and salaries were estimated using country-specific averages [28] as a percentage of the SHS workforce gross income. Total healthcare expenditure for the included countries was used from the OECD [9].

### Calculation model design

Using the collected inputs, we estimated the number of people working in school healthcare and the total salaries. To be able to compare countries, totals were converted to numbers per 1000 pupils and corrected for differences in purchasing power, lastly, all amounts were rounded to the nearest hundreds of euros. The calculation steps for both workforce and expenditure are listed in the online appendix (Supplementary Fig. 1, Online Appendix); external data used for the analyses are reported in Supplementary Table. 1, Online Appendix. These calculations were performed using Microsoft Excel [29].

## Results

### Response rate

**Fig. 1:**
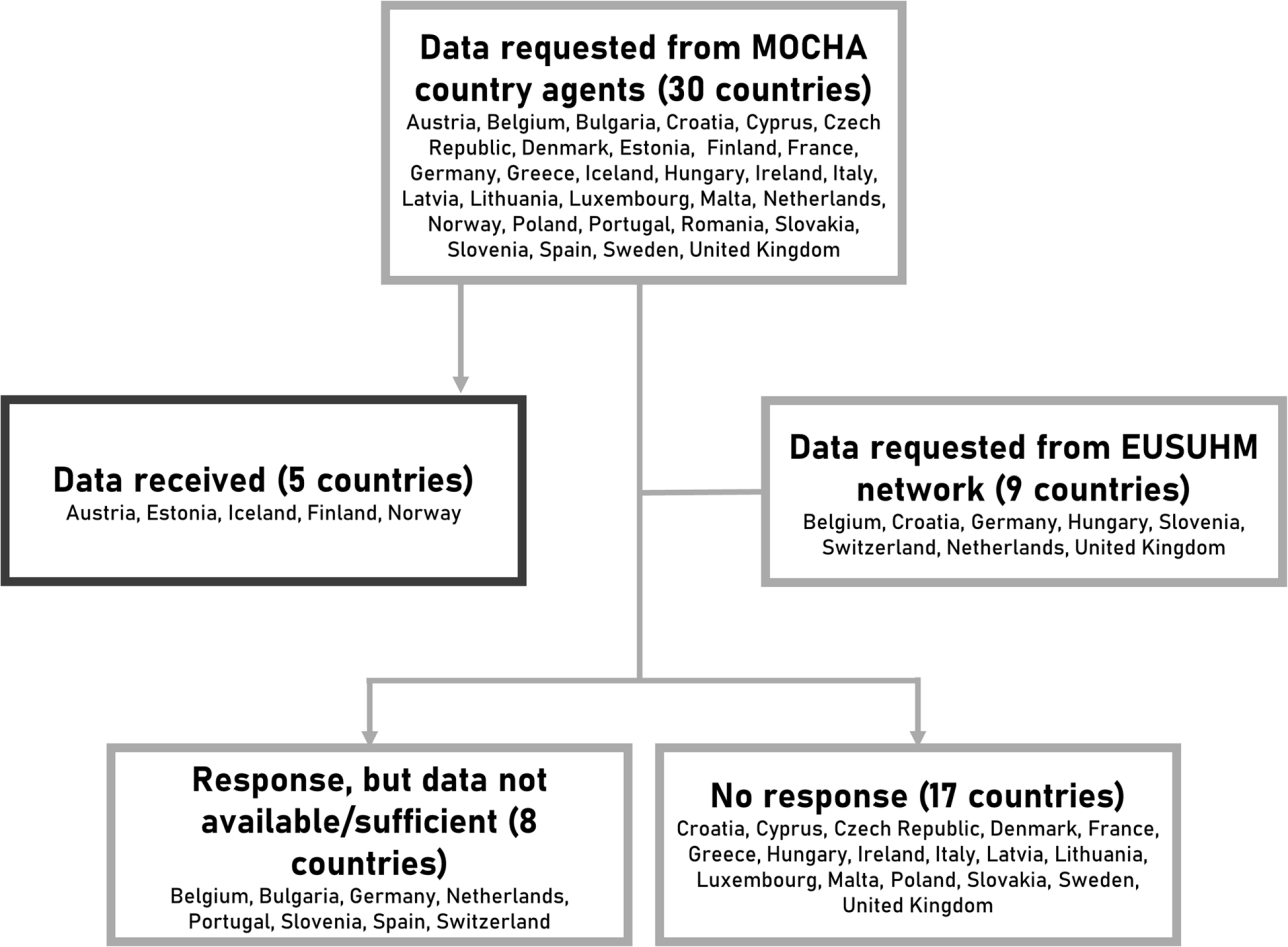
Flow chart of responding countries. *MOCHA: Models of Child Health Appraised; EUSUHM: European Union for School and University Health and Medicine*

Subsequent to sending out the questionnaire to the MOCHA country agents, we received completed questionnaires from Austria, Estonia, Finland, Iceland and Norway. The results and calculations are listed in the online appendix (Supplementary Tables 2 and 3, Online Appendix). For many countries no or insufficient data were available on the national level; this was the case for: Belgium, Bulgaria, Germany, the Netherlands, Portugal, Slovenia, Spain and Switzerland. From the other country agents, no response was received. Additionally, no complete data were received after contacting the EUSUHM network. The results are displayed in Fig. 1, all acquired data are displayed in appendix 1.

### Workforce

**Fig. 2:**
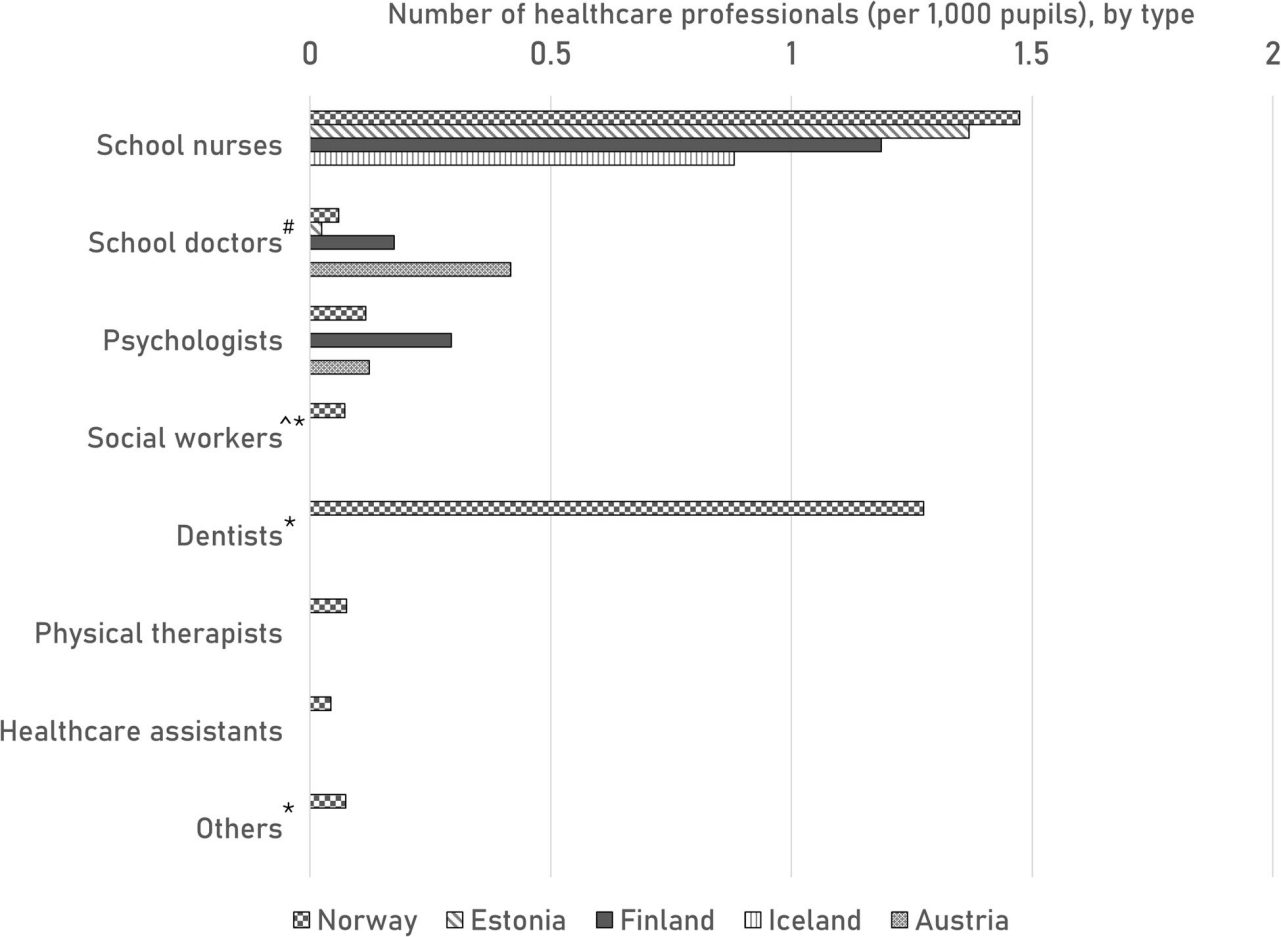
Number of school health professionals by type, per 1000 pupils. *^: indicates missing data from Finland;*^*#*^: indicates missing data from Iceland; *: indicates missing data from Austria

**Fig. 3:**
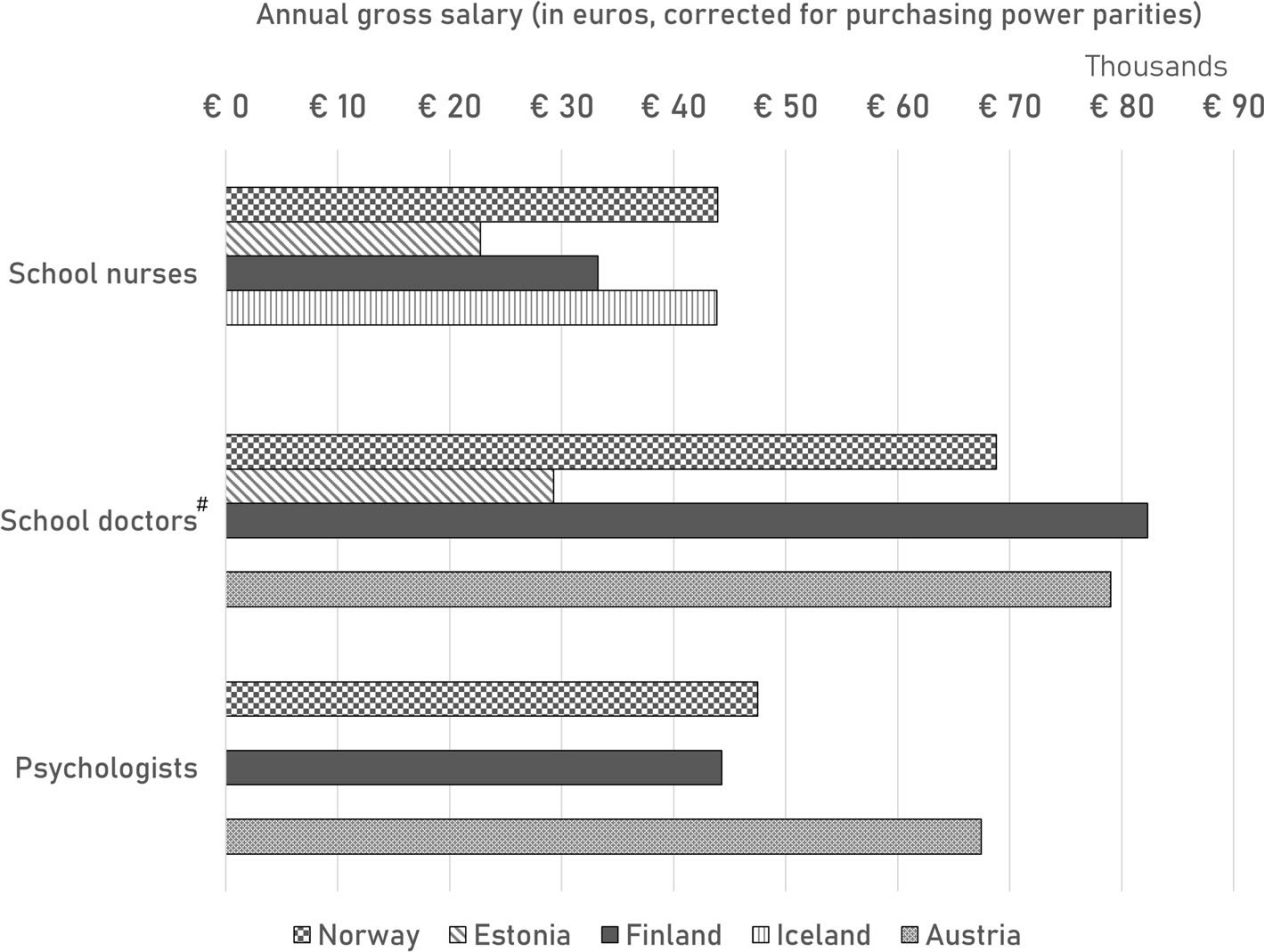
Annual salaries per school health professional function. *Only included are school nurses, school doctors and psychologists;*^*#*^*: indicates missing data from Iceland*

In Fig. 2, the number of health care professionals per 1000 pupils are displayed. Norway reports numbers on social workers, dentists, physical therapists, healthcare assistants and other SHS personnel. School nurses are reported for all included countries, except Austria. Both Norway and Estonia report around 1.4 school nurses on average per 1000 pupils; Finland 1.2 and Iceland reports 0.9. Data are missing for certain countries; no numbers are reported for certain professionals although they are part of a country’s SHS [6]: school doctors for Iceland; social workers for Finland and Austria; dentists for Austria; and others for Austria. Salaries of SHS professionals are reported in Fig. 3. In addition to the salaries displayed in Fig. 3, Norway reports salaries of €39,900 for social workers; €58,600 for dentists; €38,100 for physical therapists; €35,800 for healthcare assistants and €50,300 for others (administrators/leaders). The salary of school doctors is missing for Iceland [6].

### Expenditure

**Fig. 4:**
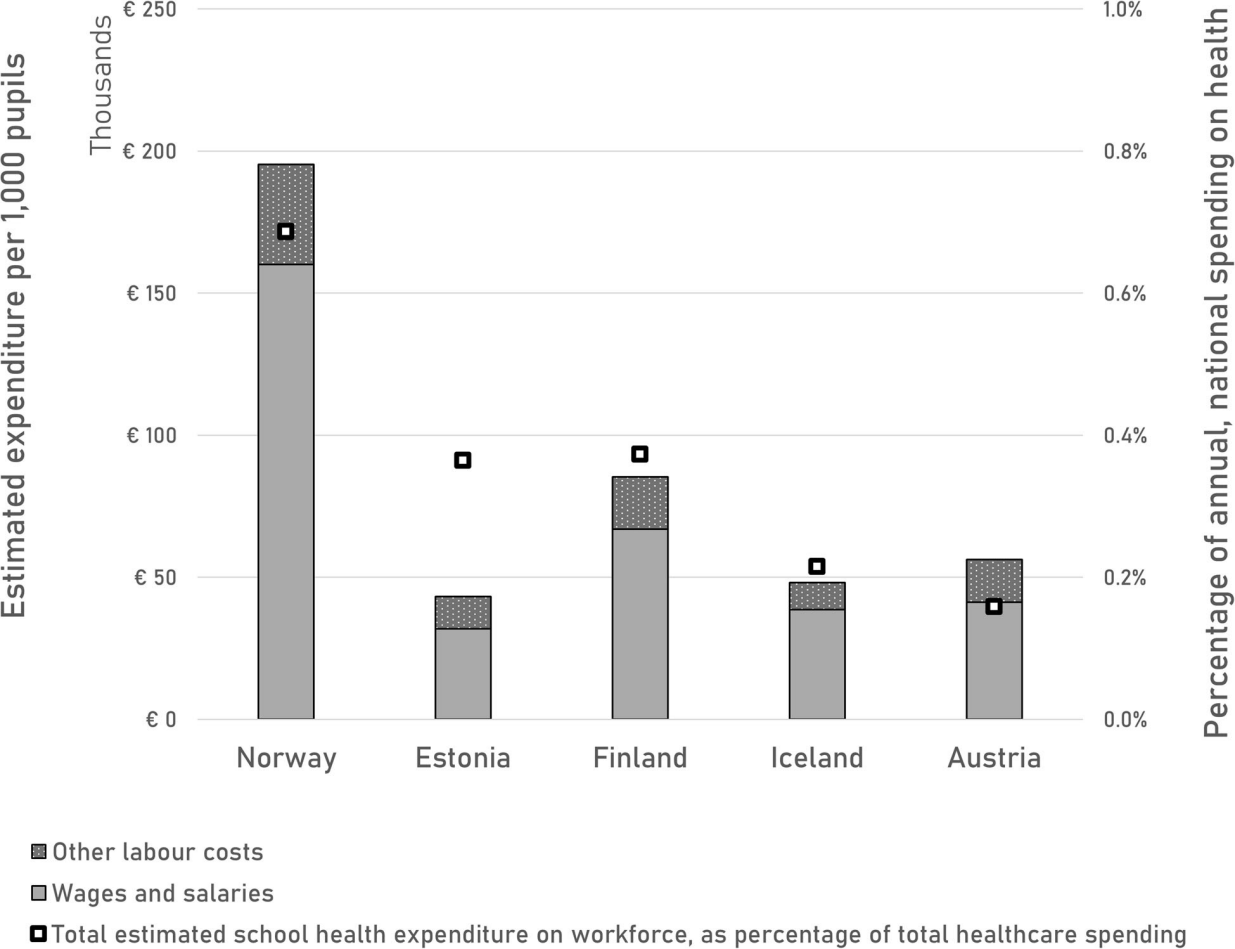
School health workforce expenditure per 1000 pupils and as a percentage of national health expenditure

The total estimated expenditure on salaries per 1000 pupils is displayed in Fig. 4, as well as the total SHS expenditure as a percentage of the national health budget on the secondary y-axis. Norway, which reports most types of SHS staff, also spends most on SHS: around €195,000 per 1000 pupils. Finland is estimated to spend around €85,000; Austria around €56,000; and for Estonia and Iceland we calculate around €45,000 per 1000 pupils. These estimations of SHS workforce expenditure range from 0.16 to 0.69% of total health expenditure for the included countries. All respondents indicate that SHS staff have a salary paid by the government.

## Discussion

Our results show estimated SHS workforce costs for five European countries, ranging from €43,100 for Estonia to €195,300 in Norway (per 1000 pupils). For Norway, Estonia, Finland and Iceland, school nurses are the main drivers of SHS expenditure, mainly due to their large numbers. In Austria, school doctors’ salaries contribute to most of SHS workforce spending, with school psychologists playing a relatively minor role due to their modest numbers.

For most countries that are included in our analysis, more health professionals are working indirectly in SHS, making it more difficult to provide estimates of SHS workforce. Examples include nurses in Austria or various health professionals collaborating closely with school nurses in health centres in Iceland. Their work description may include a role in advancing public health for children, however, they are not formally employed in school healthcare. For health systems which are tightly integrated on various levels, it may be difficult to separate the various functions and settings within the system. Many services may be provided to children which are considered a part of SHS in one country yet not in another. For example, dental care in Iceland, which is free for all children, is not considered part of SHS; or social workers who are employed by the municipality in Austria. Frequently, these differences between countries are cultural and historical; the overarching effectiveness of delivering healthcare within school services, as opposed to an alternative primary care setting, remains to be investigated further [30].

As mentioned in the introduction, we hypothesise that workforce spending is the most important driver of school health expenses*.* No expensive materials are needed, such as expensive medicine or advanced diagnostics [6]. Facility costs are limited, a room inside a school or first-line care facility will suffice. We tried to estimate some of the overhead costs by including supportive staff in the questionnaire, however, no numbers were reported by any of the country agents. Part of the overhead costs is included in the *other labour costs*, which are reported in Fig. 4. Another possible limitation of this study is the reliance on country agents to report SHS professionals and salaries, however, all country agents have experience with this type of research through the MOCHA project and were trained within this project to provide reliable information [7].

This is the first research project which examines the spending on workforce and wages of SHS personnel in European countries. We estimate this for five countries and find large differences in both workforce and workforce expenditure. However, we are careful in making policy recommendations based on these data, as the aim of this study is not to compare the functioning of SHS between different countries and the full picture regarding child health is missing, as still some data are not available. For most European countries, data on the workforce and associated expenditure remain unknown. Here, we identify a major gap in knowledge on how an important part of European health systems is financed.

From a policy perspective, we believe this lack of data may be a threat to the maintenance and further development of European school health. Within MOCHA, almost all countries indicated a shortage of staff in SHS [6], however, no data seems to be available to support this claim. Although school healthcare is considered an important part of our health systems as an entry point to target almost all children and adolescents [8], increasingly, there is a focus on quantifying healthcare decisions, e.g. by performing multi-criteria decision analyses [31, 32]. This requires standardized data on the total SHS workforce and expenditure, to place specific interventions within school healthcare in perspective and estimate the impact on the overall SHS budget. For certain activities within the health system, such as caesarean sections, MRI exams or length-of-stay for myocardial infarction, rather exact data are available, allowing healthcare professionals, researchers and policy makers to compare different regions and countries [33].

For the future, more accurate measurements of the SHS workforce, performance and expenditure need to be made. We recommend a bottom-up approach; starting on the regional level and working up from there. Consecutively, spending for specific functions (screenings, vaccination, hygienic measures etc.) within SHS can be identified and prioritized for further (cost-)effectiveness analyses. This may provide decision makers with the necessary tools to decide where to invest within healthcare provision for children with the aim of improving health for all.

## Conclusions

We estimate the spending on SHS workforce for five European countries, which is a relatively minor part of total healthcare spending (0.16 to 0.69%) in these countries. Many questions regarding SHS spending in Europe remain, due to a general lack of data on the national level.

## Supplementary information

**Additional file 1.** Online appendix. Online appendix containing additional tables (public data sources, survey results and workforce cost estimations) and a figure detailing the calculation steps.

**Additional file 2.** Questionnaire. Questionnaire sent to country agents to fill in.

## Data Availability

The datasets generated and/or analysed during the current study are available in the University of Groningen repository, https://www.rug.nl/research/portal/datasets/school-health-workforce-expenditure-across-five-countries(c1e8871f-7f6f-4ff4-ab5a-bc8328859d68).html

## Abbreviations

SHS: School Health Services
MOCHA: Models of Child Health Appraised
OECD: Organisation for Economic Co-operation and Development
EUSUHM: European Union for School and University Health and Medicine
PPP: Purchasing Power Parity
