# Supplementary material for: School health in Europe: a review of workforce expenditure across five countries
Source: BMC Health Serv Res. 2020 Mar 12;20:206. doi: 10.1186/s12913-020-05077-w (PMC7068946; doi:10.1186/s12913-020-05077-w)
Supplement: Supplementary file 1 — Additional file 1. Online appendix. Online appendix containing additional tables (public data sources, survey results and workforce cost estimations) and a figure detailing the calculation steps. [file 12913_2020_5077_MOESM1_ESM.pdf]

Online appendix

School Health in Europe: A Review of Workforce Expenditure Across Five Countries

Simon van der Pol; Maarten J. Postma; Danielle E.M.C. Jansen

Contents

Supplementary table 1 – public data sources 2

Supplementary table 2 – survey results 3

Supplementary table 3 – workforce cost estimations 4

Supplementary figure 1 – calculation steps 5

Supplementary table 1 – public data sources

	**Austria**	**Estonia**	**Finland**	**Iceland**	**Norway**
**Data year**	2018	2017	2017	2018	2016
**Population 4-18 year olds** (Eurostat)	1277309	205248	904671	67258	955476
**Conversion factor to 2018 euros, corrected for PPP** (OECD)	0.879388819	0.624720371	0.996246295	155.2012143	12.06252753
**Percentage incurred by "other labour costs"** (Eurostat)	36%	35%	28%	25%	22%
**Healthcare spending in data year, local currency (millions)** (OECD)	€39,883	€1,518	€20,621	233,780 kr	328,134 kr

PPP: purchasing power parities

Supplementary table 2 – survey results

		Austria	Estonia	Finland	Iceland	Norway
**FTEs**	School nurses	NA	281*	1073	59.23	1408
	School doctors	532^	5*	158	0	56.8
	Psychologists	157*	NA	265	0	110.4
	Social workers	NA	NA	0	0	68.8
	Dentists	NA	NA	0	0	1218
	Physical therapists	NA	NA	0	0	72.6
	Healthcare assistants	NA	NA	0	0	40.8
	Supportive staff	NA	NA	0	0	0
	Others	NA	NA	NA	NA	70.5
**Salaries**	School nurses	€ 14,208	€ 33,125	6,804,000 kr	kr 530,000	NA
	School doctors	€ 18,300	€ 81,975	NA	kr 830,000	€ 69,500
	Psychologists	NA	€ 44,125	NA	kr 573,000	€ 59,309
	Social workers	NA	€ 41,938	NA	kr 481,800	NA
	Dentists	NA	€ 77,450	NA	kr 707,160	NA
	Physical therapists	NA	NA	NA	kr 460,000	NA
	Healthcare assistants	NA	NA	NA	kr 432,000	NA
	Supportive staff	NA	NA	NA	NA	NA
	Others	NA	NA	NA	kr 607,320	NA
	Type of employmnet	Salaried	Salaried	Salaried	Salaried	Salaried
	Year	2017	2017	2018	2016	2018

*only a number of professionals was provided, not the number of full-time equivalents

^estimated based on 0.25 FTE for 600 children in the Austrian population

Supplementary table 3 – workforce cost estimations

Country	Total estimated spending on SHS workforce (% of total healthcare spending)	Estimated spending on SHS workforce, per 1,000 pupils
**Austria**	€71,817,209 (0.16%)	€56,225
**Estonia**	€8,852,043 (0.36%)	€43,129
**Finland**	€77,097,560 (0.37%)	€85,222
**Iceland**	€3,234,405 (0.21%)	€48,090
**Norway**	€186,612,220 (0.69%)	€195,308

Currencies corrected using purchasing power parities and consumer price indexes (to the year 2018)

SHS: School Health Services

Supplementary figure 1 – calculation steps

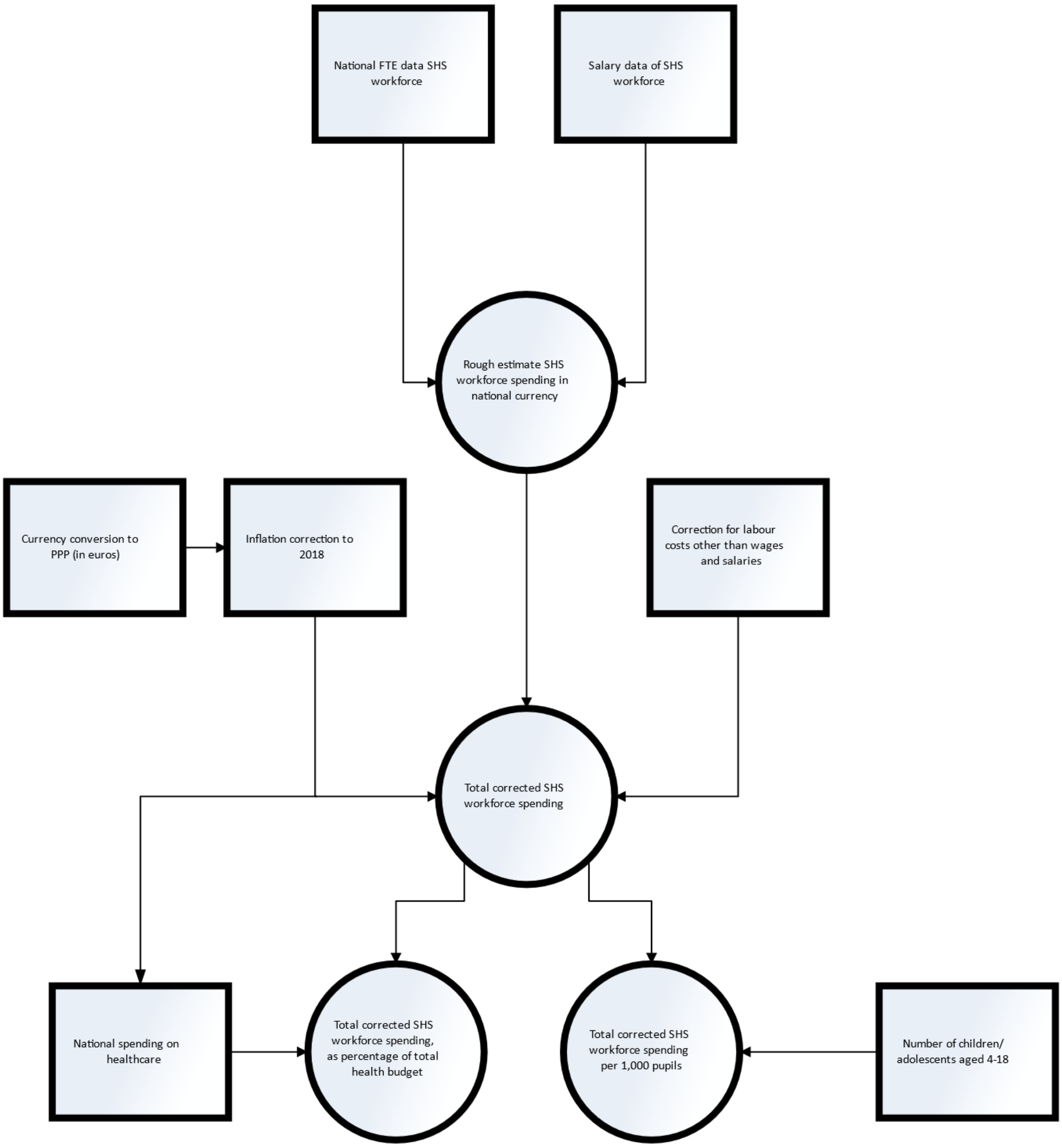

Calculation steps to get outcomes of the analysis, the squares indicate inputs and the circles indicate outcomes

FTE: full-time equivalents; SHS: School Health Services; PPP: Purchasing Power Parity
